# Hemoglobin-to-Red Cell Distribution Width Ratio as a Prognostic Marker in Decompensated Heart Failure Patients: A Prospective Observational Study

**DOI:** 10.3390/life16040551

**Published:** 2026-03-27

**Authors:** Ruxandra Maria Christodorescu, Călin Muntean, Minodora Andor, Daniel Lighezan, Adina Pop Moldovan, Andrei Blajevschi, Samuel Ardelean, Dan Darabanțiu

**Affiliations:** 1 Department V—Internal Medicine 1, “Victor Babes” University of Medicine and Pharmacy, Eftimie Murgu Square, No. 2, 300041 Timisoara, Romania; christodorescu.ruxandra@umft.ro (R.M.C.); andor.minodora@umft.ro (M.A.); dlighezan@umft.ro (D.L.); 2 Research Centre of Timisoara Institute of Cardiovascular Diseases, Gheorghe Adam St., No. 13A, 300310 Timisoara, Romania; 3 Department III-Functional Sciences—Medical Informatics and Biostatistics, “Victor Babes” University of Medicine and Pharmacy, Eftimie Murgu Square, No. 2, 300041 Timisoara, Romania; 4 Multidisciplinary Heart Research Center, Faculty of Medicine, “Victor Babes” University of Medicine and Pharmacy, Eftimie Murgu Square, No. 2, 300041 Timisoara, Romania; 5 Multidisciplinary Doctoral School, Vasile Goldis Western University of Arad, 310025 Arad, Romania; pop-modovan.adina@uvvg.ro; 6 Cardiology Department, Arad County Clinical Emergency Hospital, 310037 Arad, Romania; 7 Institute of Cardiovascular Diseases, Cardiovascular Disease Institute Timisoara, Gheorghe Adam St., No. 13A, 300310 Timisoara, Romania; blajevschiandrei@gmail.com; 8 Doctoral School, Faculty of Medicine, “Victor Babes” University of Medicine and Pharmacy, Eftimie Murgu Square, No. 2, 300041 Timisoara, Romania; samuel.ardelean@umft.ro; 9 Arad County Emergency Clinical Hospital, 310158 Arad, Romania; ddarabantiu@yahoo.com

**Keywords:** heart failure, hemoglobin, red cell distribution width, ejection fraction, prognosis, mortality, biomarker

## Abstract

Background and Methods: This prospective observational study investigated the prognostic value of the hemoglobin-to-red cell distribution width ratio (HRR) in 278 patients hospitalized with decompensated heart failure (HF). The primary endpoint was a composite of all-cause mortality or HF rehospitalization at 12 months. Multivariable Cox regression was employed to adjust for risk factors including age, sex, NT-proBNP, LVEF, and eGFR. Results: The median HRR was 0.89. During follow-up, the primary endpoint occurred in 167 (60.1%) patients. Unadjusted analysis showed a lower HRR was significantly associated with reduced event-free survival (log-rank *p* = 0.027). However, after multivariable adjustment, this association was no longer statistically significant (*p* = 0.240). Older age and male sex remained independent predictors. Conclusions: In patients with decompensated HF, a lower baseline HRR correlates with increased risk but does not maintain independent prognostic value after adjusting for powerful confounders. HRR may serve as a simple, initial marker of risk rather than an independent predictor.

## 1. Introduction

Heart failure (HF) remains a leading cause of cardiovascular morbidity and mortality worldwide, affecting over 64 million people globally [1]. Decompensated HF, the most common presentation of acute HF, is characterized by a rapid worsening of symptoms that necessitates urgent hospitalization [2]. Despite significant advances in therapeutic management, the prognosis for patients hospitalized with decompensated HF remains poor, with persistently high rates of readmission and mortality [3,4].

Accurate risk stratification is crucial for guiding clinical decision-making and optimizing therapeutic strategies in this high-risk population [5]. While established clinical parameters, imaging findings, and biomarkers such as natriuretic peptides (e.g., NT-proBNP) and cardiac troponins provide significant prognostic information, their utility can be constrained by certain limitations. For instance, the interpretation of natriuretic peptide levels is often confounded by factors such as advanced age, obesity, and particularly renal dysfunction, which is highly prevalent in the HF population and can artificially elevate NT-proBNP levels due to reduced clearance [5,6].

Parameters derived from a routine complete blood count have drawn considerable attention as potential prognostic indicators in cardiovascular diseases [7,8]. Hemoglobin is a well-established biomarker for the evaluation of anemia, and changes in hemoglobin levels may also provide indirect information about a patient’s volume status. Anemia, a frequent comorbidity in HF, is well-known to be associated with worse clinical outcomes and reduced functional capacity [9,10]. Similarly, an elevated red cell distribution width (RDW), a measure of the variability in erythrocyte size (anisocytosis), has emerged as a powerful and independent predictor of adverse outcomes in HF. High RDW is thought to reflect a combination of underlying pathological processes, including inflammation, oxidative stress, undernutrition, and ineffective erythropoiesis [11].

The hemoglobin-to-red cell distribution width ratio (HRR), a novel composite marker derived from these two parameters, has been initially proposed as a prognostic tool to assess survival in various cancers (e.g., lung, renal, liver, and colorectal) [12,13]. A lower HRR, indicating lower hemoglobin and higher RDW, reflects increased inflammation and poor prognosis, with higher mortality risk. Recent research showed that HRR may be used for prognostic information in various cardiovascular conditions [14]. The ratio theoretically integrates the impact of both anemia and increased anisocytosis, potentially providing a more holistic view of the patient’s hematological and inflammatory status. However, the clinical significance of HRR in the specific context of decompensated HF, particularly its relationship with functional status and its prognostic value independent of powerful confounders, has not been comprehensively evaluated in a prospective setting.

Therefore, this study aimed to prospectively investigate the association between baseline HRR and the 12-month risk of all-cause mortality or rehospitalization for HF in a cohort of patients hospitalized for decompensated heart failure. We also intended to explore its correlation with dynamic clinical parameters, such as changes in NYHA class and body weight during hospitalization.

## 2. Materials and Methods

### 2.1. Study Design and Population

This prospective observational registry enrolled consecutive patients hospitalized with a primary diagnosis of decompensated HF at two cardiology departments (Arad County Hospital and Municipal Hospital Timisoara) between January 2021 and December 2022. The study was conducted in accordance with the Declaration of Helsinki [15], and the protocol was approved by the institutional ethics committees of both participating centers. Written informed consent was obtained from all participants.

Inclusion criteria were (1) age ≥18 years and (2) a diagnosis of acute decompensated HF according to the 2021 European Society of Cardiology (ESC) guidelines. Exclusion criteria included: (1) life expectancy less than 12 months due to non-cardiovascular causes; (2) active hematological disorders or known malignancy; (3) blood transfusion within the preceding 3 months; (4) acute coronary syndrome within the 30 days prior to admission or (5) severe valvular disease requiring imminent surgical or percutaneous intervention. Patients did not receive oral or IV iron supplementation during hospitalization. Therefore, data on oral or intravenous iron supplementation during the 12-month outpatient follow-up were not systematically collected. No patient had an implantable cardioverter-defibrillator (ICD) or cardiac resynchronization therapy (CRT) device at the time of inclusion.

### 2.2. Data Collection and Measurements

Comprehensive clinical data were collected upon hospital admission, including demographic characteristics, medical history, cardiovascular risk factors, and etiology of HF. Data on guideline-directed medical therapy (GDMT) for HF were recorded at the time of hospital discharge. Clinical status, including New York Heart Association (NYHA) functional class and body weight, was assessed at both admission and discharge. The difference in weight between these two points was considered a marker of decongestion.

Venous blood samples were drawn within 24 h of admission. A complete blood count, including hemoglobin (g/dL) and RDW (%), was performed using an automated hematology analyzer (Sysmex XN-3000, Kobe, Japan). The HRR was calculated as the ratio of hemoglobin to RDW. N-terminal pro-B-type natriuretic peptide (NT-proBNP) was measured using a standardized electrochemiluminescence immunoassay (Roche Diagnostics, Cobas 6000 analyzer, Rotkreuz, Switzerland). Estimated Glomerular Filtration Rate (eGFR) was calculated using the Chronic Kidney Disease Epidemiology Collaboration (CKD-EPI) formula and was used in all subsequent analyses to ensure a more accurate assessment of renal function.

### 2.3. Echocardiographic Assessment

All patients underwent a comprehensive transthoracic echocardiography within 48 h of admission. Left ventricular ejection fraction (LVEF) was calculated using the biplane Simpson’s method. In accordance with current ESC guidelines, patients were categorized into three groups: HF with reduced ejection fraction (HFrEF; LVEF < 40%), HF with mildly reduced ejection fraction (HFmrEF; LVEF 40–49%), and HF with preserved ejection fraction (HFpEF; LVEF ≥ 50%).

### 2.4. Follow-Up and Endpoints

The primary endpoint of the study was a composite of all-cause mortality or the first rehospitalization for HF within 12 months of discharge. Follow-up data were collected through scheduled outpatient visits or structured telephone interviews.

### 2.5. Statistical Analysis

Statistical analysis was conducted using MedCalc^®^ Statistical Software version 22.016, Microsoft Office Excel Professional Plus 2021, R version 4.2.0, and JASP version 0.19.3. Continuous variables were expressed as mean ± standard deviation (SD) or median and interquartile range (IQR) based on their distribution, which was assessed using the Shapiro–Wilk test. Categorical variables were presented as frequencies and percentages. Group comparisons were performed using Student’s *t*-test or Mann–Whitney U test for continuous variables and the chi-square test for categorical variables. Analysis of variance (ANOVA) was used for multiple group comparisons.

Receiver operating characteristic (ROC) curve analysis was conducted to evaluate the ability of HRR, RDW, and hemoglobin to predict the primary composite endpoint, and the area under the curve (AUC) was calculated. The optimal cutoff value for HRR was determined using the Youden index (J = sensitivity + specificity − 1). Kaplan–Meier survival analysis with the log-rank test was used to compare event-free survival rates between patient groups stratified by the cohort median HRR value. Multivariable Cox proportional hazards regression analysis was performed to identify independent predictors of the primary endpoint. The final model included HRR, age, sex, LVEF, log-transformed NT-proBNP, and eGFR (CKD-EPI). The proportional hazards assumption was verified for all models. A two-sided *p*-value < 0.05 was considered statistically significant.

NYHA functional class at discharge was not included in the primary multivariable model because it represents a post-baseline variable reflecting treatment response, and its inclusion could introduce overadjustment bias by conditioning on an intermediate variable on the causal pathway between baseline HRR and the outcome. A sensitivity analysis including NYHA class at discharge is reported separately. To evaluate additive discriminative value beyond the base model (age, sex, LVEF, log[NT-proBNP], eGFR), Net Reclassification Improvement (NRI) and Integrated Discrimination Improvement (IDI) analyses were performed as described by Pencina et al. As a further sensitivity analysis, cause-specific Cox regression models were constructed for each component of the composite endpoint separately. For the rehospitalization endpoint, a Fine–Gray competing risks model was additionally fitted with death as the competing event.

## 3. Results

### 3.1. Baseline Characteristics

A total of 278 patients who met the inclusion criteria were enrolled in the final analysis (Table 1). The mean age was 69.4 ± 12.0 years, and 169 patients (60.8%) were male. The etiology of heart failure was ischemic in 66 patients (23.7%) and non-ischemic in 212 patients (76.3%). The cohort was predominantly composed of patients with HFrEF (*n* = 193, 69.4%), followed by HFpEF (*n* = 46, 16.5%) and HFmrEF (*n* = 39, 14.0%). At admission, 99.3% of patients were in advanced NYHA class (III or IV). The mean weight was 82.5 ± 20.9 kg (*n* = 278). Following in-hospital treatment and significant decongestion, evidenced by a mean weight loss of 4.4 ± 5.4 kg, a substantial clinical improvement was observed, with 57.3% of patients being in NYHA class I or II at discharge. The median HRR for the entire cohort was 0.89 (IQR: 0.75–1.00).

During the 12-month follow-up, 23 of the 278 enrolled patients (8.3%) were lost to follow-up (low HRR group: *n* = 5; high HRR group: *n* = 18). These patients were censored at their last known contact date in all time-to-event analyses. The number at risk at baseline reflects the 255 patients with confirmed events or complete follow-up data. Atrial fibrillation was documented in 147 patients (52.9%), with no significant difference between HRR groups (54.3% vs. 50.7%, *p* = 0.568).

Patients were stratified into two groups based on the median HRR value. The baseline characteristics of the study cohort, stratified by HRR group, are presented in Table 1. Patients in the low HRR group were slightly older, had a lower prevalence of male sex, and exhibited a more adverse biomarker profile, including significantly higher NT-proBNP levels, lower eGFR, lower hemoglobin, and higher RDW.

### 3.2. HRR Distribution and Correlations

**Figure 1:**
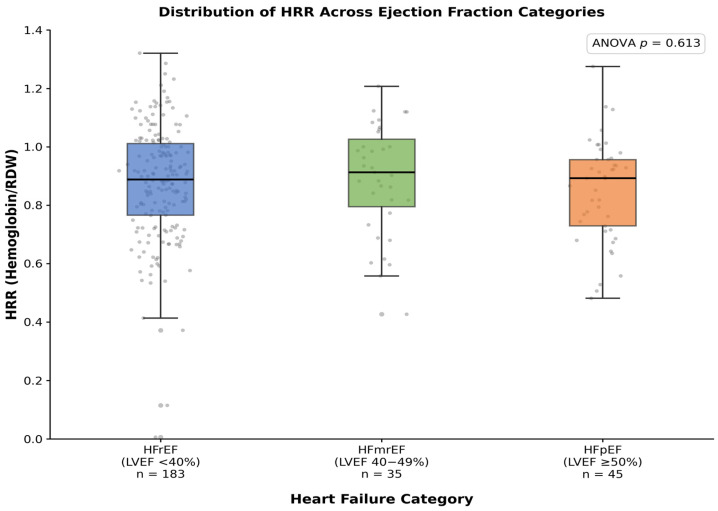
Distribution of HRR Across Ejection Fraction Categories. Box plots showing the distribution of HRR in patients with heart failure with reduced ejection fraction (HFrEF), mildly reduced ejection fraction (HFmrEF), and preserved ejection fraction (HFpEF). The boxes represent the interquartile range (IQR) with the median indicated by the horizontal line. The overlaid gray circles represent individual patient data points.

**Figure 2:**
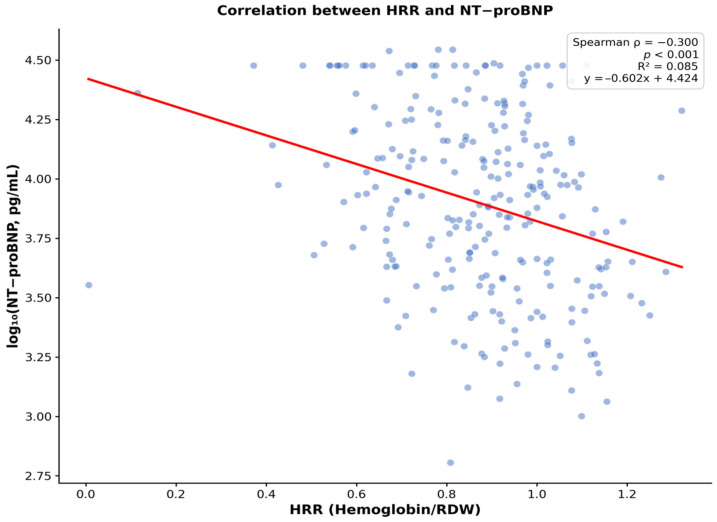
Correlation between HRR and NT-proBNP. Scatter plot showing a significant negative correlation between HRR and log-transformed NT-proBNP levels (Spearman’s ρ = −0.300, *p* < 0.001). The red line represents the linear regression fit.

There was no statistically significant difference in HRR values across the three ejection fraction categories (HFrEF: 0.88 ± 0.20; HFmrEF: 0.89 ± 0.19; HFpEF: 0.85 ± 0.17; *p* = 0.613 by ANOVA), suggesting that the prognostic implications of HRR may not be specific to any particular HF phenotype (Figure 1). A weak but statistically significant inverse correlation was observed between HRR and NYHA functional class at discharge (Spearman’s ρ = −0.152, *p* = 0.017). Furthermore, HRR showed a significant negative correlation with log-transformed NT-proBNP levels (Spearman’s ρ = −0.300, *p* < 0.001) (Figure 2) and a positive correlation with eGFR (Spearman’s ρ = 0.274, *p* < 0.001), indicating that a lower HRR is associated with more severe cardiac dysfunction and worse renal function.

### 3.3. Prognostic Value of HRR

Over the 12-month follow-up period, the primary composite endpoint occurred in 167 patients (60.1%), consisting of 74 deaths (26.6%) and 137 rehospitalizations for HF (49.3%).

ROC curve analysis was performed to assess the prognostic utility of HRR for the composite endpoint. The AUC for HRR was 0.554 (95% CI: 0.48–0.63), indicating no statistically significant discriminative ability. For comparison, the AUC for RDW alone was 0.539 and for hemoglobin alone was 0.536.

**Figure 3:**
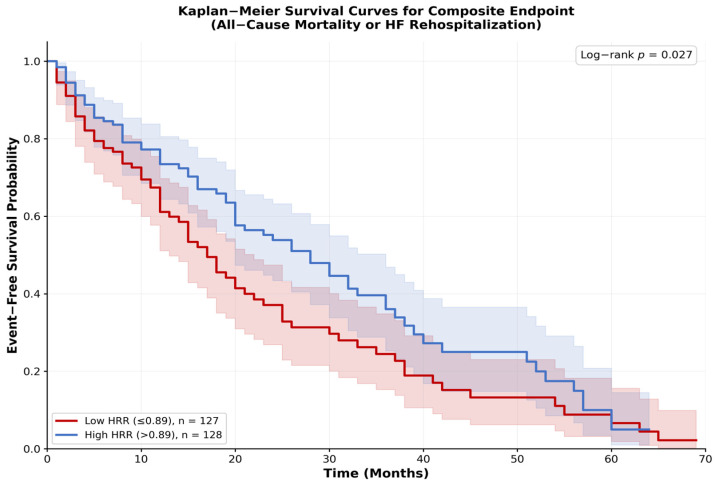
Kaplan–Meier Survival Curves for the Composite Endpoint. Event-free survival probability for the composite endpoint of all-cause mortality or HF rehospitalization, stratified by the median HRR value.

Kaplan–Meier survival analysis, performed by stratifying patients into two groups based on the cohort median HRR of 0.89, showed that patients with a low HRR had a significantly lower 12-month event-free survival rate for the composite endpoint compared to those with a high HRR (log-rank *p* = 0.027) (Figure 3).

### 3.4. Multivariable Cox Regression Analysis

We performed a multivariable Cox proportional hazards regression analysis to determine if HRR was an independent predictor of outcome. In the unadjusted model, a higher HRR was significantly associated with a reduced risk of the composite endpoint (HR 0.27, 95% CI: 0.12–0.60, *p* = 0.001).

**Figure 4:**
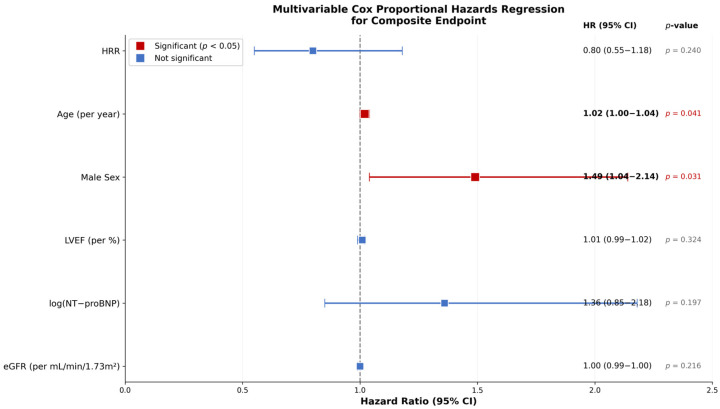
Forest Plot of Multivariable Cox Proportional Hazards Regression. Hazard ratios and 95% confidence intervals for the primary composite endpoint. The model was adjusted for age, sex, LVEF, log-transformed NT-proBNP, and eGFR. Statistically significant variables (*p* < 0.05) are highlighted in red and bold text.

However, after adjusting for established prognostic markers including age, sex, LVEF, log-transformed NT-proBNP, and eGFR, the association between HRR and the composite endpoint was no longer statistically significant (adjusted HR 0.80, 95% CI: 0.55–1.18, *p* = 0.240). In the final model, only older age and male sex remained independently associated with adverse outcomes. The results of the multivariable analysis are detailed in Table 2 and visualized in Figure 4.

For sensitivity analysis, the multivariable Cox regression model was repeated with diabetes mellitus (DM) added as an additional covariate. DM was prevalent in 105 patients (37.8%) and was numerically more frequent in the low HRR group (43.2% vs. 32.9%, *p* = 0.110). In the extended model, DM was independently associated with the composite endpoint (HR 1.52, 95% CI: 1.08–2.14, *p* = 0.017). Importantly, the inclusion of DM did not materially alter the primary finding: the adjusted HR for HRR changed minimally (from 0.80 to 0.78), and HRR remained non-significant as an independent predictor (*p* = 0.240). These results reinforce the robustness of the primary analysis and confirm that the prognostic information captured by HRR is not confounded by the presence of diabetes mellitus.

To evaluate additive discriminative value, NRI and IDI analyses were performed. The continuous NRI for HRR added to the base model (age, sex, LVEF, log[NT-proBNP], eGFR) was 0.163 (95% CI: −0.088 to 0.414, *p* = 0.204), and the IDI was 0.005 (95% CI: −0.009 to 0.018, *p* = 0.490), indicating no significant incremental reclassification or discrimination improvement. The C-statistic increased marginally from 0.641 (base model) to 0.652 (base model + HRR), a non-significant difference (ΔC = 0.011). These findings confirm that HRR does not meaningfully improve risk classification beyond established markers.

In a sensitivity analysis including NYHA functional class at discharge as an additional covariate (*n* = 243), HRR remained non-significant in the adjusted model (HR 0.26, 95% CI: 0.10–0.67, *p* = 0.005). NYHA class at discharge was independently associated with the composite endpoint (HR 1.43, 95% CI: 1.02–2.01, *p* = 0.040). Older age (HR 1.02, *p* = 0.050) and male sex (HR 1.54, *p* = 0.020) remained significant predictors. The model concordance improved marginally (C = 0.67).

Cause-specific Cox regression models yielded consistent results. For all-cause mortality (62 events), the adjusted HR for HRR was 0.34 (95% CI: 0.09–1.23, *p* = 0.100); for HF rehospitalization (127 events, censoring deaths), the adjusted HR was 0.34 (95% CI: 0.13–0.87, *p* = 0.025). In the Fine–Gray subdistribution hazard model for rehospitalization with death as the competing event, results were directionally consistent with the cause-specific analysis. These supplementary analyses support the interpretation that HRR at admission does not independently predict either component of the composite endpoint after multivariable adjustment.

## 4. Discussion

Risk assessment in patients with decompensated heart failure (HF) is a central component of modern HF management, as it directly informs clinical decision-making, level of care, therapeutic intensity, and prognosis estimation. In acute settings, it functions both as a triage tool and as a dynamic monitoring framework [2]. In our prospective study of patients hospitalized with decompensated HF, the principal finding was that a lower baseline HRR at admission was associated with adverse outcomes in unadjusted analyses. However, after adjusting for established risk factors such as age, sex, LVEF, NT-proBNP, and eGFR, it does not remain an independent predictor of all-cause mortality or HF rehospitalization at 12 months. This nuanced finding highlights the complexity of risk stratification in HF and suggests that the prognostic information provided by HRR may be largely captured by other powerful markers.

An important methodological consideration is the plain discordance between the significant unadjusted association of HRR with outcomes (log-rank *p* = 0.027; univariate Cox HR 0.27, *p* = 0.001) and the near-chance ROC performance (AUC = 0.554, 95% CI: 0.48–0.63). These findings are not contradictory but reflect fundamentally distinct analytical questions. Kaplan–Meier and Cox regression are the primary tools for time-to-event endpoints, incorporating the temporal dimension of event occurrence and censored observations—both critical when the endpoint is time to death or first HF rehospitalization. The Cox model directly addresses this study’s central research question: whether HRR is independently associated with prognosis after multivariable adjustment. Because this study was designed as an associative investigation, the Cox regression hierarchy—from significant unadjusted to non-significant adjusted association (HR 0.80, *p* = 0.240)—constitutes the primary finding.

The assessment of weight dynamics between admission and discharge offers a nuanced view of the patient’s decongestion status. In our cohort, patients were predominantly overweight or obese, with a mean body weight of 82.5 ± 20.9 kg. The change in weight reflects distinct pathophysiological processes. While rapid weight loss during hospitalization (4.4 ± 5.4 kg) primarily shows effective diuretic-induced decongestion, the baseline nutritional status, inferred from BMI, is still a critical contextual factor. Overweight and obese patients with decompensated HF exist in a state of high-grade, chronic systemic inflammation, which is a key driver of disease progression, particularly HFpEF. This inflammatory state is largely fueled by dysfunctional adipose tissue (especially visceral and epicardial fat), which releases proinflammatory cytokines (TNF-IL-6) and acts as an “immune organ” in response to overnutrition [16]. A low HRR, indicative of inflammation and potential iron deficiency, may therefore identify a subgroup of patients in whom weight loss represents a successful decongestion but also a higher pro-inflammatory state in which adipose tissue plays a role. In our cohort, the mean weight loss of 4.4 kg as a decongestion marker was not significantly different in the HRR groups (*p* = 0.96).

The potential mechanisms linking a low HRR to adverse outcomes in HF are likely multifactorial, although no inflammatory or iron metabolism biomarkers were measured in our study. Chronic inflammation, a cornerstone of HF pathophysiology, is thought to be driven by cytokines like interleukin-6 (IL-6) and tumor necrosis factor-alpha (TNF-α), which stimulate hepatic production of hepcidin [17,18]. Elevated hepcidin may lead to functional iron deficiency by trapping iron within macrophages and enterocytes, thereby contributing to anemia (low hemoglobin) [19,20]. Simultaneously, these inflammatory cytokines are thought to suppress erythroid progenitor cells in the bone marrow, leading to ineffective erythropoiesis and an increased RDW [21]. We hypothesize that a low HRR may reflect a dysfunctional hematopoietic system under the strain of chronic inflammation and dysregulated iron metabolism—a mechanistic interpretation that requires direct validation through measurement of inflammatory and iron metabolism biomarkers (including IL-6, hepcidin, ferritin, and transferrin saturation) in future prospective studies [22].

Additionally, hepatic congestion and metabolic abnormalities contribute to the complex pathophysiological environment of decompensated HF [23,24]. In our cohort, liver transaminases were mildly elevated (median AST 31.0 U/L, ALT 24.0 U/L), consistent with the hepatic congestion phenotype characteristic for decompensated HF. Total cholesterol levels were notably low (148.0 ± 43.9 mg/dL), particularly in the low HRR group (139.4 ± 44.0 vs. 153.9 ± 42.7 mg/dL, *p* = 0.011), consistent with the well-described “lipid paradox” in decompensated heart failure, where lower cholesterol levels reflect greater catabolic burden and are paradoxically associated with worse outcomes [25]. These metabolic markers provide additional context for interpreting HRR in the decompensated HF setting.

Our finding that HRR at admission loses its independent prognostic value after adjustment for NT-proBNP and eGFR is important. While NT-proBNP is a gold-standard marker of myocardial atrial wall stress, its levels can be influenced by multiple factors, including renal function, age, and obesity [6,26]. The strong correlation between HRR and both NT-proBNP and eGFR suggests that these markers share common pathophysiological pathways. The prognostic information provided by HRR at admission, discharge, and during follow-up in our cohort needs to be further investigated in future research.

Our results are consistent with several recent studies that have shown mixed findings regarding the independent prognostic value of HRR. Chen et al. [27] demonstrated that HRR at admission was a predictor of clinical outcomes and diuretic response in acute HF patients. Kanzaki et al. showed the prognostic impact of HRR in patients after acute decompensated HF [28]. Similarly, Li et al. found that HRR was associated with in-hospital mortality in chronic HF patients [29]. However, the degree to which HRR provides independent prognostic information beyond established markers varies across studies, likely reflecting differences in patient populations, endpoints, and statistical approaches [30].

While the simplicity and universal availability of HRR from routine complete blood counts remain attractive features, our finding that HRR lacks independent predictive value after multivariable adjustment, combined with its near-chance discriminative performance (AUC 0.554) and non-significant NRI/IDI results, precludes any recommendation for its use as a standalone triage or prognostic tool in current clinical practice. The association observed in unadjusted analyses should be regarded as a hypothesis-generating signal rather than evidence supporting clinical implementation. Whether HRR may contribute incremental value within composite risk scores in resource-limited settings where natriuretic peptides are unavailable warrants investigation in larger, multicenter validation cohorts.

It is also important to mention that, across the world, there are factors that limit the use of recommendations from the current guidelines, such as differences in access to the healthcare resources, in the local infrastructure, and in practices [31]. Therefore, new global heart failure guidelines were developed that aim to be applicable to all cardiovascular care worldwide. The results of our study may provide an easy-to-perform marker of initial HF severity, helping to stratify patients presenting with decompensated HF in emergency departments.

### Limitations

Our study has several limitations. First, as an observational registry, we can only identify associations and cannot establish causality. Second, while the total sample size was adequate for the primary analysis, it was insufficient for robust subgroup analyses stratified by ejection fraction. Third, a significant limitation is the lack of data on specific iron deficiency parameters (e.g., transferring saturation) and inflammatory cytokines, which would have allowed for a more direct exploration of the underlying pathophysiological mechanisms. Fourth, data on SGLT2 inhibitor use were limited in this cohort. Fifth, Kaplan–Meier and Cox regression are optimally suited for our time-to-event endpoint—preserving temporal information and censored observations. Finally, HRR was measured only at a single time point upon admission; evaluating its dynamic changes during hospitalization could provide further prognostic information.

## 5. Conclusions

In this prospective cohort of patients with decompensated heart failure, a lower baseline hemoglobin-to-red cell distribution width ratio (HRR) at admission was associated with an increased risk of adverse outcomes at 12 months across the spectrum of ejection fraction (EF) in unadjusted analyses. However, this association was not maintained after multivariable adjustment for major confounders, particularly NT-proBNP and renal function (eGFR). HRR may represent a simple and readily available marker of systemic vulnerability, reflecting inflammation and hematopoietic dysfunction in patients hospitalized for decompensated HF; however, it did not demonstrate prognostic value independent of established risk factors in this cohort. Its clinical utility may lie in the initial risk assessment in settings such as emergency departments, particularly in smaller hospitals, where it could help identify patients with decompensated HF who may require more aggressive management.

## Figures and Tables

**Table 1:** Baseline characteristics of the study cohort, stratified by median HRR.

Characteristic	All Patients (*N* = 278)	Low HRR (≤0.89) (*n* = 132)	High HRR (>0.89) (*n* = 146)	*p*-Value
Demographics				
Age, years	69.4 ± 12.0	70.8 ± 11.8	68.2 ± 12.0	0.077
Male Sex, *n* (%)	169 (60.8)	70 (53.0)	99 (67.8)	0.017
Heart Failure Profile				
LVEF, %	33.6 ± 11.8	33.2 ± 11.9	33.9 ± 11.7	0.616
HFrEF (LVEF < 40%), *n* (%)	193 (69.4)	93 (70.5)	100 (68.5)	0.722
HFmrEF (LVEF 40–49%), *n* (%)	39 (14.0)	18 (13.6)	21 (14.4)	
HFpEF (LVEF ≥ 50%), *n* (%)	46 (16.5)	21 (15.9)	25 (17.1)	
NYHA Class III–IV at discharge, *n* (%)	111 (42.7)	62 (50.4)	49 (36.0)	0.017
Laboratory Parameters				
Hemoglobin, g/dL	13.1 ± 1.9	12.0 ± 1.7	14.2 ± 1.4	<0.001
RDW, %	15.1 ± 2.0	16.3 ± 2.0	13.9 ± 1.2	<0.001
NT-proBNP, pg/mL *	8240 (4033–15,019)	9330 (5169–20,221)	6897 (3298–11,736)	<0.001
eGFR, mL/min/1.73 m^2^	61.5 ± 24.8	56.1 ± 25.3	66.5 ± 23.4	<0.001
Hepatic and Metabolic Parameters				
AST, U/L *	31.0 (20.8–48.0)	30.0 (20.0–46.5)	32.0 (21.5–50.0)	0.443
ALT, U/L *	24.0 (14.0–53.2)	21.5 (13.0–47.0)	25.0 (16.5–62.0)	0.123
Total Cholesterol, mg/dL	148.0 ± 43.9	139.4 ± 44.0	153.9 ± 42.7	0.011
Comorbidities				
Diabetes mellitus, *n* (%)	105 (37.8)	57 (43.2)	48 (32.9)	0.110
Atrial fibrillation, *n* (%)	147 (52.9)	70 (54.3)	68 (50.7)	0.568
GDMT at Discharge				
Beta-Blocker, *n* (%)	244 (87.8)	115 (87.1)	129 (88.4)	0.730
MRA, *n* (%)	226 (81.3)	107 (81.1)	119 (81.5)	0.932
ACEi/ARB, *n* (%)	127 (45.7)	58 (45.0)	58 (43.3)	0.784
ARNI, n (%)	66 (23.7)	29 (22.5)	37 (27.6)	0.337
SGLT2 inhibitor, *n* (%)	94 (33.8)	42 (32.6)	49 (36.6)	0.494
Loop diuretic, *n* (%)	268 (96.4)	121 (93.8)	132 (98.5)	0.046
Ivabradine, *n* (%)	31 (11.2)	17 (13.2)	12 (9.0)	0.274
Digoxin, *n* (%)	64 (23.0)	28 (21.7)	33 (24.6)	0.575
Statin, *n* (%)	115 (41.4)	55 (42.6)	57 (42.5)	0.987
Oral anticoagulant, *n* (%)	155 (55.8)	68 (52.7)	77 (57.5)	0.439
Antiplatelet, *n* (%)	66 (23.7)	34 (26.4)	31 (23.1)	0.545

* Data are presented as mean ± SD, median (IQR), or *n* (%). Abbreviations:; HRR, hemoglobin-to-RDW ratio; LVEF, left ventricular ejection fraction; HFrEF, heart failure with reduced ejection fraction; HFmrEF, heart failure with mildly reduced ejection fraction; HFpEF, heart failure with preserved ejection fraction; NYHA, New York Heart Association; RDW, red cell distribution width; NT-proBNP, N-terminal pro-B-type natriuretic peptide; eGFR, estimated glomerular filtration rate; GDMT, guideline-directed medical therapy; MRA, mineralocorticoid receptor antagonist. AST, aspartate aminotransferase; ALT, alanine aminotransferase; ACEi, angiotensin-converting enzyme inhibitor; ARB, angiotensin receptor blocker; ARNI, angiotensin receptor-neprilysin inhibitor; SGLT2 inhibitor, sodium-glucose co-transporter 2 inhibitor.

**Table 2:** Multivariable Cox proportional hazards analysis for the primary composite endpoint.

Variable	Hazard Ratio (HR)	95% CI	*p*-Value
HRR	0.80	0.55–1.18	0.240
Age (per year)	1.02	1.00–1.04	0.041
Male Sex	1.49	1.04–2.14	0.031
LVEF (per %)	1.01	0.99–1.02	0.324
log(NT-proBNP)	1.36	0.85–2.18	0.197
eGFR (per mL/min)	1.00	0.99–1.00	0.216

Abbreviations: HRR, hemoglobin-to-RDW ratio; LVEF, left ventricular ejection fraction; NT-proBNP, N-terminal pro-B-type natriuretic peptide.

## Data Availability

The data presented in this study is available on request from the corresponding author. The data is not publicly available due to privacy restrictions regarding patient information, in accordance with the General Data Protection Regulation (GDPR).

## Abbreviations

The following abbreviations are used in this manuscript:

AHA	American Heart Association
ANOVA	Analysis of Variance
AUC	Area Under the Curve
BMI	Body Mass Index
CHF	Congestive Heart Failure
CHS	Cardiovascular Health Study
CI	Confidence Interval
ESC	European Society of Cardiology
HF	Heart Failure
HFmrEF	Heart Failure with Mildly Reduced Ejection Fraction
HFpEF	Heart Failure with Preserved Ejection Fraction
HFrEF	Heart Failure with Reduced Ejection Fraction
HGB	Hemoglobin
HR	Hazard Ratio
HRR	Hemoglobin-to-Red Cell Distribution Width Ratio
IQR	Interquartile Range
*JCM*	*Journal of Clinical Medicine*
LVEF	Left Ventricular Ejection Fraction
NT-proBNP	N-terminal pro-B-type natriuretic peptide
NYHA	New York Heart Association
OR	Odds Ratio
RDW	Red Cell Distribution Width
ROC	Receiver Operating Characteristic
SBP	Systolic Blood Pressure
SD	Standard Deviation

## References

[1] Savarese G., Lund L.H. (2017). Global Public Health Burden of Heart Failure. Card. Fail. Rev..

[2] McDonagh T.A., Metra M., Adamo M., Baumbach A., Böhm M., Burri H., Butler J., Čelutkienė J., Chioncel O., Cleland J.G.F. (2021). 2021 ESC Guidelines for the diagnosis and treatment of acute and chronic heart failure: Developed by the Task Force for the diagnosis and treatment of acute and chronic heart failure of the European Society of Cardiology (ESC) with the special contribution of the Heart Failure Association (HFA) of the ESC. Eur. Heart J..

[3] Mebazaa A., Yilmaz M.B., Levy P., Ponikowski P., Peacock W.F., Laribi S., Ristic A.D., Lambrinou E., Masip J., Riley J.P. (2015). Recommendations on Pre-Hospital & Early Hospital Management of Acute Heart Failure: A Consensus Paper from the Heart Failure Association of the European Society of Cardiology, the European Society of Emergency Medicine and the Society of Academic Emergency Medicine. Eur. J. Heart Fail..

[4] Abraham W.T., Fonarow G.C., Albert N.M., Stough W.G., Gheorghiade M., Greenberg B.H., O’COnnor C.M., Sun J.L., Yancy C.W., Young J.B. (2008). Predictors of In-Hospital Mortality in Patients Hospitalized for Heart Failure: Insights From the Organized Program to Initiate Lifesaving Treatment in Hospitalized Patients with Heart Failure (OPTIMIZE-HF). J. Am. Coll. Cardiol..

[5] Bayes-Genis A., Docherty K.F., Petrie M.C., Januzzi J.L., Mueller C., Anderson L., Bozkurt B., Butler J., Chioncel O., Cleland J.G. (2023). Practical algorithms for early diagnosis of heart failure and heart stress using NT-proBNP: A clinical consensus statement from the Heart Failure Association of the ESC. Eur. J. Heart Fail..

[6] Januzzi J.L., Chen-Tournoux A.A., Christenson R.H., Doros G., Hollander J.E., Levy P.D., Nagurney J.T., Nowak R.M., Pang P.S., Patel D. (2018). N-Terminal Pro–B-Type Natriuretic Peptide in the Emergency Department: The ICON-RELOADED Study. J. Am. Coll. Cardiol..

[7] Salvagno G.L., Sanchis-Gomar F., Picanza A., Lippi G. (2015). Red blood cell distribution width: A simple parameter with multiple clinical applications. Crit. Rev. Clin. Lab. Sci..

[8] Felker G.M., Allen L.A., Pocock S.J., Shaw L.K., McMurray J.J.V., Pfeffer M.A., Swedberg K., Wang D., Yusuf S., Michelson E.L. (2007). Red Cell Distribution Width as a Novel Prognostic Marker in Heart Failure. Data From the CHARM Program and the Duke Databank. J. Am. Coll. Cardiol..

[9] Groenveld H.F., Januzzi J.L., Damman K., van Wijngaarden J., Hillege H.L., van Veldhuisen D.J., van der Meer P. (2008). Anemia and Mortality in Heart Failure Patients: A Systematic Review and Meta-Analysis. J. Am. Coll. Cardiol..

[10] Horwich T.B., Fonarow G.C., Hamilton M.A., MacLellan W.R., Borenstein J. (2002). Anemia is associated with worse symptoms, greater impairment in functional capacity and a significant increase in mortality in patients with advanced heart failure. J. Am. Coll. Cardiol..

[11] Dai Y., Konishi H., Takagi A., Miyauchi K., Daida H. (2014). Red cell distribution width predicts short- and long-term outcomes of acute congestive heart failure more effectively than hemoglobin. Exp. Ther. Med..

[12] Lai T., Liang Y., Guan F., Hu K. (2025). Trends in hemoglobin-to- red cell distribution width ratio and its prognostic value for all-cause, cancer, and cardiovascular mortality: A nationwide cohort study. Sci. Rep..

[13] Chi G., Lee J.J., Montazerin S.M., Marszalek J. (2022). Prognostic value of hemoglobin-to-red cell distribution width ratio in cancer: A systematic review and meta-analysis. Biomark. Med..

[14] Li M., Li H., Zhong W., Wang S., Liu R., Cheng H., Li L., Wei Q., Wang L. (2025). Hemoglobin-to-Red Cell Distribution Width Ratio Was Associated with Cardiovascular Diseases and Death. J. Clin. Med..

[15] Rickham P.P. (1964). Human Experimentation: Code of Ethics of W.M.A. Br. Med. J..

[16] Sabbah M.S., Fayyaz A.U., De Denus S., Felker G.M., Borlaug B.A., Dasari S., Carter R.E., Redfield M.M. (2020). Obese-Inflammatory Phenotypes in Heart Failure with Preserved Ejection Fraction. Circ. Heart Fail..

[17] García-Escobar A., Lázaro-García R., Goicolea-Ruigómez J., Pizarro G., Jurado-Román A., Moreno R., Cabrera J.Á. (2024). Red Blood Cell Distribution Width as a Biomarker of Red Cell Dysfunction Associated with Inflammation and Macrophage Iron Retention: A Prognostic Marker in Heart Failure and a Potential Predictor for Iron Replacement Responsiveness. Card. Fail. Rev..

[18] Liang L., Huang L., Zhao X., Zhao L., Tian P., Huang B., Feng J., Zhang J., Zhang Y. (2022). Prognostic value of RDW alone and in combination with NT-proBNP in patients with heart failure. Clin. Cardiol..

[19] Silverberg D.S., Wexlerb D., Iaina A., Schwartz D. (2008). The Role of Correction of Anaemia in Patients with Congestive Heart Failure: A Short Review. Eur. J. Heart Fail..

[20] Jankowska E.A., Rozentryt P., Witkowska A., Nowak J., Hartmann O., Ponikowska B., Borodulin-Nadzieja L., Banasiak W., Polonski L., Filippatos G. (2010). Iron deficiency: An ominous sign in patients with systolic chronic heart failure. Eur. Heart J..

[21] Pascual-Figal D.A., Bonaque J.C., Redondo B., Caro C., Manzano-Fernandez S., Sánchez-Mas J., Garrido I.P., Valdes M. (2009). Red Blood Cell Distribution Width Predicts Long-Term Outcome Regardless of Anaemia Status in Acute Heart Failure Patients. Eur. J. Heart Fail..

[22] Anker S.D., Comin Colet J., Filippatos G., Willenheimer R., Dickstein K., Drexler H., Lüscher T.F., Bart B., Banasiak W., Niegowska J. (2009). Ferric Carboxymaltose in Patients with Heart Failure and Iron Deficiency. N. Engl. J. Med..

[23] Ambrosy A.P., Gheorghiade M., Bubenek S., Vinereanu D., Vaduganathan M., Macarie C., Chioncel O., Romanian Acute Heart Failure Syndromes (RO-AHFS) study investigators (2013). The predictive value of transaminases at admission in patients hospitalized for heart failure: Findings from the RO-AHFS registry. Eur. Heart J. Acute Cardiovasc. Care.

[24] van Deursen V.M., Damman K., Hillege H.L., van Beek A.P., van Veldhuisen D.J., Voors A.A. (2010). Abnormal Liver Function in Relation to Hemodynamic Profile in Heart Failure Patients. J. Card. Fail..

[25] Greene S.J., Vaduganathan M., Lupi L., Ambrosy A.P., Mentz R.J., Konstam M.A., Nodari S., Subacius H.P., Fonarow G.C., Bonow R.O. (2013). Prognostic Significance of Serum Total Cholesterol and Triglyceride Levels in Patients Hospitalized for Heart Failure with Reduced Ejection Fraction (from the EVEREST Trial). Am. J. Cardiol..

[26] Ibrahim N.E., Januzzi J.L. (2018). Established and emerging roles of biomarkers in heart failure. Circ. Res..

[27] Chen H., Zhen Z., Dong Y., Liu C., Dong B., Xue R. (2024). Hemoglobin to red cell distribution width ratio: A predictor of clinical outcome and diuretic response in patients with acute heart failure. Int. J. Cardiol..

[28] Kanzaki Y., Minamisawa M., Motoki H., Suzuki S., Okuma Y., Kanai M., Machida K., Kimura K., Ueki Y., Yoshie K. (2025). Prognostic Impact of the Ratio of Hemoglobin to Red Blood Cell Distribution Width in Patients after Acute Decompensated Heart Failure. Intern. Med..

[29] Li Y., Xu C., Qin Z., Ge L. (2024). Relationship Between the Hemoglobin-to-Red Cell Distribution Width Ratio and in-Hospital Mortality in Patients with Chronic Heart Failure. Vasc. Health Risk Manag..

[30] Xu X., Yang R., Yin Y., Zhu Y., Si J., Xu Y. (2025). Association of hemoglobin-to-red blood cell distribution width ratio with mortality in critically Ill patients with heart failure and acute kidney injury: Insights from the MIMIC-IV database. BMC Cardiovasc. Disord..

[31] Chopra V., Khan M.S., Abdelhamid M., Abraham W.T., Amir O., Anker S.D., Atherton J.J., Bacal F., von Bardeleben R.S., Brito D. (2025). iCARDIO Alliance Global Implementation Guidelines on Heart Failure 2025. Heart Lung Circ..

